# Control of Dynamic Positioning System with Disturbance Observer for Autonomous Marine Surface Vessels

**DOI:** 10.3390/s21206723

**Published:** 2021-10-10

**Authors:** Mirosław Tomera, Kamil Podgórski

**Affiliations:** 1 Department of Ship Automation, Gdynia Maritime University, 81-87 Morska St., 81-225 Gdynia, Poland; 2 Doctoral School, Gdynia Maritime University, 81-87 Morska St., 81-225 Gdynia, Poland; k.podgorski@sd.umg.edu.pl

**Keywords:** dynamic positioning, backstepping control, disturbance observer, marine autonomous surface vessel

## Abstract

The main goal of the research is to design an efficient controller for a dynamic positioning system for autonomous surface ships using the backstepping technique for the case of full-state feedback in the presence of unknown external disturbances. The obtained control commands are distributed to each actuator of the overactuated vessel via unconstrained control allocation. The numerical hydrodynamic model of *CyberShip I* and the model of environmental disturbances are applied to simulate the operation of the ship control system using the time domain analysis. Simulation studies are presented to illustrate the effectiveness of the proposed controller and its robustness to external disturbances.

## 1. Introduction

Extensive development work is currently underway on the concept of the Maritime Autonomous Surface Ship (MASS), which requires new solutions in many areas: law, economics, guidance, control and navigation [1,2,3]. Automated navigation tasks require the further development of high-level control systems; in particular, with respect to such issues as path planning and collision avoidance [4,5,6,7,8,9,10,11,12,13,14,15]. However, the basic issue in the research on developing autonomous marine surface vessels is motion control. Model-based control is used to steer and dynamically position the ship. This type of determining control algorithm became the most common approach in the beginning of the 1960s, when such techniques as the Linear Quadratic-Gaussian (LQG) and other approaches determined in the state space were used. The models used in the design of model-based control systems depend on control objectives. These targets can be roughly divided into low-speed positioning and high-speed steering [16]. The first target, called dynamic positioning (DP), involves station keeping, position mooring and dynamic tracking control at low speed [17,18,19,20,21]. High-speed steering includes automatic heading control [22,23,24,25,26,27,28,29,30], high speed position tracking [31,32,33,34,35,36], path following [37,38,39,40], roll motion control [41,42,43] and formation control [44,45].

In view of the maneuvering difficulties caused by the weight of a ship, it is not an easy task to improve the quality of navigation, especially for ships moving at low speed (called dynamic positioning). Designing an efficient dynamic positioning (DP) system for a marine vessel is a challenging practical problem. The performance and robustness of the DP system is essential for the success of the mission. In dynamic positioning systems, the main goal is to keep the marine vessel in a steady position and at a constant heading (direction) in the horizontal plane or to follow the target trajectory using only hull-mounted thrusters. The first generation of dynamic positioning systems comes from the early 1960s, when drilling began to be performed at very great depths. The first vessel equipped with a dynamic positioning system was *Eureka*, owned by Shell Oil Company, which entered into operation in 1961 [46]. Currently, dynamic positioning systems are used on various types of ships to perform many marine tasks, such as hydrographic surveys, marine construction, wreck research and geodesy. In the offshore oil and gas industry, many tasks can only be performed with the assistance of DP systems. This refers to the operation of service vessels, rigs and drilling vessels, shuttle tankers, cable and pipe laying units and floating production storage and offloading (FPSO) units.

The first implemented dynamic positioning systems made use of PID (Proportional–Integral–Derivative) controllers. To counteract the excessive activity of thrusts associated with wave-caused hull motion, the controllers used cut-off filters in a cascade arrangement with low-pass filters [47]. The improvement in the quality of control system operation took place after the application of more advanced control techniques based on the optimal control theory and the Kalman filter theory [48,49,50,51]. The major disadvantage of this approach was that the kinetic equations of motion had to be linearized for certain conditions. For each linearized equation, new gains were computed for the Kalman filter and for coupling, and then these gains were modified online by gain scheduling. In the 1990s, introducing nonlinear observers and feedback control theory to the designs of dynamic positioning systems resulted in the removal of assumptions related to linearization [52,53,54].

The further development of control algorithms used in dynamic positioning systems was associated with the emergence of alternative control strategies such as robust control [55,56,57,58,59,60,61,62,63], modal control [64,65], adaptive control [66] and model predictive control [46,67,68,69]. Other solutions for control systems were related to the developments taking place in non-linear control [70,71] using methods such as backstepping [72,73,74,75,76,77,78], dynamic surface control [79,80], active direct surface control [81], nonlinear PID control [18,82,83], port-Hamiltonian framework [84] and sliding mode control [85,86,87]. A hybrid DP system using supervisory switching control logic to change between the bank of controllers and observers was also proposed [88,89,90].

The dynamic positioning systems presented in some works applied tools used for modeling artificial intelligence, such as fuzzy systems [91,92,93,94,95,96,97], artificial neural networks [98,99,100,101,102,103] and neuro-fuzzy systems [104,105]. An overview of selected research works related to the technological progress in the design of dynamic positioning control systems was presented in [19,21].

In dynamic positioning systems, multivariable controllers usually determine the commanded forces and torque, which must then be generated by (obtained from) thrusters installed on the ship. For overactuated marine vessels, control allocation is a vital part of the DP system. Improper allocation may lead to degraded control performance, lower energy efficiency and the increased wear and tear of the actuators. There is a rich literature regarding control allocation for marine surface vessels, commonly referred to as thrust allocation [106,107,108,109,110,111,112,113,114,115,116,117,118,119,120]. In-depth reviews of the literature are given in [121,122].

A vessel operating in the ocean is subjected to disturbances caused by waves, wind and sea currents that cause the vessel to deviate from its desired position and direction. Hence, disturbance damping is one of the key problems in the design of DP control. The ocean disturbance can be divided into low-frequency (LF) disturbance caused by second-order waves, sea currents and wind and wave frequency (WF) disturbance caused by first-order waves. The low-frequency disturbance causes the ship to drift, while the wave frequency disturbance causes it to oscillate. The compensation of wave frequency disturbances would wear out the marine actuators and increase fuel consumption. On the other hand, there is no need to compensate for WF disturbances because they cause only oscillatory movements of the ship [69]. These motions should be filtered off by wave filtering algorithms from the vessel position and heading measurements before passing them to the DP control system. Several wave filtering techniques have been proposed [123,124,125,126]. Therefore, in this article, only the low-frequency components of environmental disturbances are considered in the DP control design.

The control objective in this paper is to design a ship motion control algorithm in dynamic positioning using the backstepping method with a disturbance observer of unmeasured disturbances affecting the ship’s hull.

## 2. Formulation of the Problem of Steering the Ship at Low Speed

**Figure 1:**
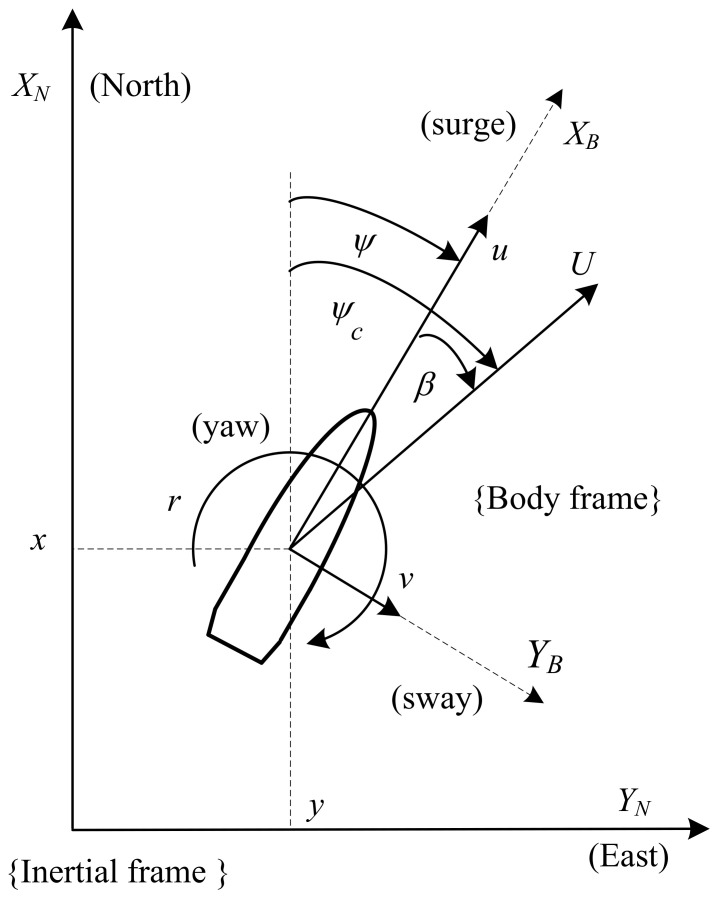
Coordinate systems and variables used to describe the ship motion.

The motion of a ship sailing on the water surface in the horizontal plane is analyzed in three degrees of freedom. This motion is described using two coordinate systems, limited to two dimensions: the inertial frame ($X_{N}$, $Y_{N}$) associated with the sea map and the body-fixed reference frame ($X_{B}$,$Y_{B}$) associated with the moving ship (Figure 1). Physical quantities assumed as state variables for the ship moving in the horizontal plane can be grouped into two vectors: $\eta ={\left[x,y,\psi \right]}^{T}$ and $\nu ={\left[u,v,r\right]}^{T}$, where (*x*, *y*) are the coordinates of ship’s position, *ψ* is the ship’s heading, (*u*, *v*) are the linear velocity components of ship’s motion in the surge and sway directions, and *r* is the yaw rate [127].

The velocity vector determined in the inertial frame ($X_{N}$, $Y_{N}$) is related to that determined in the body-fixed reference frame ($X_{B}$, $Y_{B}$) by the following kinematic relationship:

(1) $$\dot{\eta }=R\left(\psi \right)\nu$$

where $R\left(\psi \right)\in R^{3\times 3}$ is the rotation matrix by angle ψ, determined from the relationship

(2) $$R\left(\psi \right)=\left[\begin{array}{ccc} cos(\psi ) & -sin(\psi ) & 0\\ sin(\psi ) & cos(\psi ) & 0\\ 0 & 0 & 1 \end{array}\right]$$

The properties of the rotation matrix given by Formula (2) are as follows

(3) $$∥R(\psi )∥=1,\;\;\;R^{-1}\left(\psi \right)=R^{T}\left(\psi \right),\;\;\;\frac{d}{dt}R^{T}\left(\psi \right)=-rSR^{T}\left(\psi \right)$$

where $S\in R^{3\times 3}$ is the skew-symmetric matrix

(4) $$S=\left[\begin{array}{ccc} 0 & -1 & 0\\ 1 & 0 & 0\\ 0 & 0 & 0 \end{array}\right],\;\;\;S=-S^{T}$$

The mathematical model of the dynamics of a ship sailing on the surface of the sea and ocean in the presence of environmental disturbances is described as follows [127]:

(5) $$M\dot{\nu }+C\left(\nu \right)\nu +D\left(\nu \right)\nu =\tau +R^{T}\left(\psi \right)b=\tau +\tau_{d}$$

where $M\in R^{3\times 3}$ is the inertia matrix, $C\in R^{3\times 3}$ is the matrix of Coriolis and centripetal terms, $D\in R^{3\times 3}$ is the damping matrix, and $\tau ={\left[\tau_{x},\tau_{y},\tau_{n}\right]}^{T}$ is the input control vector consisting of the surge force $\tau_{x}$, sway force $\tau_{y}$ and yaw moment $\tau_{n}$ produced by propellers and thrusters installed in the ship’s hull. Unmodeled external low frequency (LF) forces and moments due to wind, currents and waves are connected together into an Earth-fixed constant (or slowly varying) bias term $b\left(t\right)={\left[b_{1}\left(t\right),b_{2}\left(t\right),b_{3}\left(t\right)\right]}^{T}$. Here, it is assumed that the changing rate of the bias is bounded,

(6) $$∥\dot{b}\left(t\right)∥\leq C_{d}<\infty$$

where $C_{d}$ is a nonnegative constant. The above assumption is reasonable because the environmental energy applied to the vessel is limited.

In Equation (5), $\tau_{d}$ represents external disturbances acting on the vessel in the body-fixed reference frame, given as

$$\tau_{d}=R^{T}\left(\psi \right)b$$

The inertia matrix $M\in R^{3\times 3}$, which includes the hydrodynamic added inertia, can be written as [127]

(7) $$M=\left[\begin{array}{ccc} m_{11} & 0 & 0\\ 0 & m_{22} & m_{23}\\ 0 & m_{32} & m_{33} \end{array}\right]=\left[\begin{array}{ccc} m-X_{\dot{u}} & 0 & 0\\ 0 & m-Y_{\dot{v}} & mx_{G}-Y_{\dot{r}}\\ 0 & mx_{G}-N_{\dot{v}} & I_{z}-N_{\dot{r}} \end{array}\right]$$

where *m* is the vessel mass, $I_{z}$ is the moment of inertia about the fixed *z*-axis of the vessel, and $X_{\dot{u}}$, $Y_{\dot{v}}$, $Y_{\dot{r}}$, $N_{\dot{v}}$ and $N_{\dot{r}}$ are hydrodynamic derivatives. Zero-frequency masses are added to the surge, sway and yaw due to accelerations along the relevant axes. For control applications, which are restricted to LF motions, the wave frequency independence of the added inertia (zero wave frequency) can be assumed. This implies that $\dot{M}=0$.

The matrix of Coriolis and centripetal terms has the form

(8) $$C=\left[\begin{array}{ccc} 0 & 0 & -m_{22}v-m_{23}r\\ 0 & 0 & m_{11}u\\ m_{22}v+m_{23}r & -m_{11}u & 0 \end{array}\right]$$

For a straight-line stable vessel, $D\in R^{3\times 3}$ is a positive damping matrix due to linear wave drift and laminar skin friction. The linear damping matrix is defined as [127]

(9) $$D=\left[\begin{array}{ccc} -X_{u} & 0 & 0\\ 0 & -Y_{v} & -Y_{r}\\ 0 & -N_{v} & -N_{r} \end{array}\right]$$

## 3. Control Algorithms in the Dynamic Positioning System

The control objective in this paper is to design a DP control system for a ship with unknown time-varying disturbances, so that the vessel’s actual position (*x*, *y*) and heading converge to the desired values $\eta_{d}={\left[x_{d},y_{d},\psi_{d}\right]}^{T}$.

**Assumption** **1.** *The desired smooth reference signal $\eta_{d}$ is bounded and has bounded first ${\dot{\eta }}_{d}$ and second ${\ddot{\eta }}_{d}$ derivatives. This means that the functions describing the ship’s position and direction, as well as their derivatives of the first and second order, are limited*.

**Figure 2:**
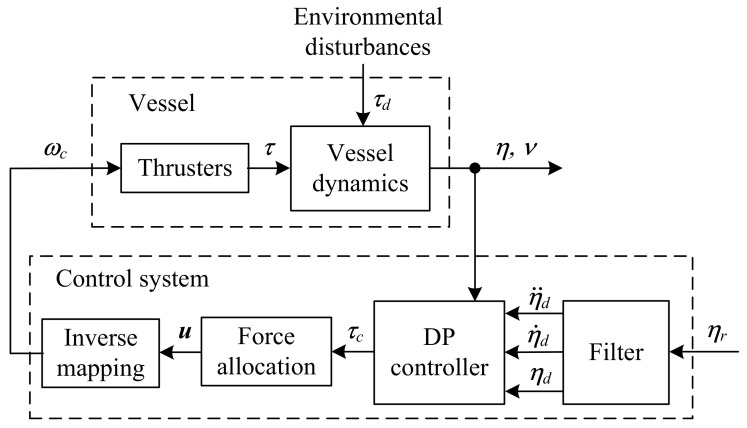
Block diagram of dynamic positioning control system.

The considered movement of the vessel is executed at low speed in the system shown in Figure 2. The input to this control system is the vector $\eta_{r}$ with reference coordinates of the ship’s position and direction. The smooth and bounded desired trajectories $\eta_{d}$ with their first ${\dot{\eta }}_{d}$ and second-order ${\ddot{\eta }}_{d}$ derivatives needed for the controller were generated by a second-order filter:

(10) $$G_{fi}\left(s\right)=\frac{\omega_{ni}^{2}}{s^{2}+2\zeta_{i}\omega_{ni}s+\omega_{ni}^{2}}\;\;i=1,2,3.$$

where the reference $\eta_{r}$ is the operator input, $\zeta_{i}$ is the relative damping ratio, and $\omega_{ni}$ is the natural frequency. Notice that

(11) $$\lim_{t\to \infty }\eta_{d}\left(t\right)=\eta_{r}$$

and ${\dot{\eta }}_{d}$ and ${\ddot{\eta }}_{d}$ are smooth and bounded derivatives for steps in $\eta_{r}$.

The vessel is assumed to be overactuated. The trajectory of motion of such a vessel depends on the operation of all actuators installed in its hull. The controller’s task is to determine the forces and moments to be applied to the ship’s hull. This also requires the use of an appropriate system for the distribution of forces and torque determined by the controller.

### 3.1. Backstepping Method with Disturbance Observer

The control algorithm used in the dynamic positioning system was derived using the backstepping method and assuming that the entire plant state vector is known. The vector of control forces $\tau_{c}\left(t\right)$ was designed in such a way as to ensure that the state variables in vectors η(t) and ν(t) remain constrained and that the position and course are asymptotically convergent to their set constant values $\eta \left(t\right)\to \eta_{d}\left(t\right)$ with ν(t)≈0 for t≥0. The classic method of backstepping is described in [72]. The desired signals required for control are represented by the given position and direction vector $\eta_{d}={\left[x_{d},y_{d},\psi_{d}\right]}^{T}$ and its first ${\dot{\eta }}_{d}$ and second ${\ddot{\eta }}_{d}$ derivatives. It is assumed that all desired signals related to the ship position ($x_{d}$, $y_{d}$) and heading $\psi_{d}$ are limited.

The control deviations related to the given position and direction vector $\eta_{d}$ and the velocity vector ν were defined as

(12) $$z_{1}=\eta -\eta_{d}$$

(13) $$z_{2}=\nu -\vartheta_{1}$$

where $\vartheta_{1}\in R^{3}$ is the stabilizing function, which is the desired virtual control. Determining the two-sided derivative from Equation (12), and substituting relation (1) and that determined from Equation (13) into this derivative, we obtain

(14) $${\dot{z}}_{1}=\dot{\eta }-{\dot{\eta }}_{d}=R\left(\psi \right)\nu -{\dot{\eta }}_{d}=R\left(\psi \right)\left(z_{2}+\vartheta_{1}\right)-{\dot{\eta }}_{d}$$

The stabilizing function $\vartheta_{1}$ was assumed as

(15) $$\vartheta_{1}=R^{-1}\left(\psi \right)\left(-K_{1}z_{1}+{\dot{\eta }}_{d}\right)$$

where $K_{1}=K_{1}^{T}>0$ is the diagonal positive definite gain matrix. The stabilizing function $\vartheta_{1}$, which is the desired virtual control for vector ν, was determined in relation to the Lyapunov function in the form $V_{1}=0.5z_{1}^{T}z_{2}$. Substituting relation (15) into Equation (14) and using the relation $R\left(\psi \right)R^{-1}\left(\psi \right)=I$, we obtain

(16) $${\dot{z}}_{1}=-K_{1}z_{1}+R\left(\psi \right)z_{2}$$

Determining the two-sided derivative from Equation (13) and substituting the relation determined from Equation (5) into it, we obtain

(17) $${\dot{z}}_{2}=\dot{\nu }-{\dot{\vartheta }}_{1}=M^{-1}\left[-C\left(\nu \right)\nu -D\nu +\tau +R^{T}\left(\psi \right)b\right]-{\dot{\vartheta }}_{1}$$

where $\dot{\vartheta_{1}}$, determined based on Equation (15), takes the form

(18) $${\dot{\vartheta }}_{1}=-rSR^{T}\left(\psi \right)\left(-K_{1}z_{1}+{\dot{\eta }}_{d}\right)+R^{T}\left(\psi \right)\left(-K_{1}{\dot{z}}_{1}+{\ddot{\eta }}_{d}\right)$$

With regard to the Lyapunov function in the form $V_{2}=V_{1}+0.5z_{2}^{T}Mz_{2}$, the following control law was adopted:

(19) $$\tau_{c}=C\left(\nu \right)\nu +D\nu -MK_{2}z_{2}-MR^{T}\left(\psi \right)z_{1}+M{\dot{\vartheta }}_{1}-R^{T}\left(\psi \right)\hat{b}$$

where $K_{2}=K_{2}^{T}>0$ is the positively defined gain matrix, and the vector $\hat{b}$ contains estimates of the parameters of the external bias term b describing external disturbances acting on the vessel. The disturbance observer for the bias vector b was constructed using the exponential convergent observer from [128]

(20) $$\hat{b}=R\left(\psi \right)\left[\theta +K_{0}M\nu \right]$$

(21) $$\dot{\theta }=-K_{0}\theta -K_{0}\left[-C\left(\nu \right)\nu -D\nu +\tau_{c}+K_{0}M\nu \right]$$

where $\hat{b}\left(t\right)={\left[{\hat{b}}_{1}\left(t\right),{\hat{b}}_{2}\left(t\right),{\hat{b}}_{3}\left(t\right)\right]}^{T}$ is the estimate of the bias term, $K_{0}$ is the 3 × 3 positive definite symmetric observer gain matrix, and θ is the 3 × 1 intermediate auxiliary vector.

Considering the ship dynamics given by Formula (5) and the desired trajectory $\eta_{d}$, which is smooth and limited, the information about all ship states x is provided. In this case, the control law is described by Formula (19). The law of adaptation of unknown parameters related to environmental disturbances is described by Formula (20) and has zero values as initial conditions. The entered design parameters $K_{0}=K_{0}^{T}>0$, $K_{1}=K_{1}^{T}>0$, $K_{2}=K_{2}^{T}>0$ are positively defined. These conditions mean that the entire closed control system is stable, and consequently the signals $z_{1}$ and $z_{2}$ have finite values.

### 3.2. Nonlinear PID Controller

The controller used to compare the obtained results of simulation tests was the nonlinear PID controller for DP systems, described in detail in [18]. The algorithm of this controller is given by the formula

(22) $$\tau_{PID}=MR^{-1}\left(\psi \right)\left[{\dot{\nu }}_{d}-K_{P}\eta_{e}-K_{I}\xi_{e}-K_{D}\nu_{e}-{\dot{\psi }}_{e}R\left(\psi \right)S\nu \right]+C\left(\nu \right)\nu +D\nu$$

where R is the rotation matrix (2), S is the skew-symmetric matrix (4), M is the inertia matrix (7), C(ν) is the Coriolis and centripetal matrix (8), D is the matrix of hydrodynamic damping (9), and $K_{P}$, $K_{I}$ and $K_{D}$ are the matrix gains of the nonlinear PID controller. In this algorithm, the position and orientation errors $\eta_{e}$ are determined in the inertial frame ($X_{N}$, $Y_{N}$), while the velocity error $\nu_{e}$ and the acceleration error ${\dot{\nu }}_{e}$ are determined in the body-fixed reference frame ($X_{B}$, $Y_{B}$) associated with the moving ship

(23) $$\eta_{e}=\eta -\eta_{d}$$

(24) $$\nu_{e}=\nu -\nu_{d}=\nu -R^{T}\left(\psi \right){\dot{\eta }}_{d}$$

(25) $${\dot{\nu }}_{e}=\dot{\nu }-{\dot{\nu }}_{d}=\dot{\nu }-\dot{\psi_{e}}S^{T}R^{T}\left(\psi \right){\dot{\eta }}_{d}-R^{T}\left(\psi \right){\ddot{\eta }}_{d}$$

(26) $${\dot{\psi }}_{e}=\dot{\psi }-{\dot{\psi }}_{d}=r-{\dot{\psi }}_{d}$$

(27) $${\dot{\xi }}_{e}=\eta_{e}$$

The gain matrices of the nonlinear PID controller are positively defined: $K_{P}=K_{P}^{T}>0$, $K_{I}=K_{I}^{T}>0$, and $K_{D}=K_{D}^{T}>0$.

### 3.3. Unconstrained Control Allocation

The vectors $\tau_{c}$ or $\tau_{PID}$ of the desired forces and moments determined by the DP controllers were divided by the allocation system into the commanded values for the actuators installed in the ship’s hull (Figure 2). It is assumed that the ship is fitted with *q* propulsion devices located at positions

(28) $$l\left(i\right)=\left[l_{i,x},l_{i,y}\right]$$

where $l_{i,x}$, $l_{i,y}$ are the distances of the propulsion devices from the origin of the coordinate system associated with the moving ship. These devices can provide the thrust force $T_{i}$ in the direction defined by the angle $\alpha_{i}$. The azimuth thrusters have the angle $\alpha_{i}$ fixed in a certain direction and produce the following contributions to the generalized forces acting on the ship [120]:

(29) $$T_{xi}=T_{i}cos\left(\alpha_{i}\right)$$

(30) $$T_{yi}=T_{i}sin\left(\alpha_{i}\right)$$

(31) $$N\left(i\right)=T_{i}L_{i}$$

where $L_{i}=l_{i,x}sin\left(\alpha_{i}\right)-l_{i,y}cos\left(\alpha_{i}\right)$.

The sum of the generalized forces acting on the ship’s hull from all installed propellers is given by the formula

(32) $$\tau_{c}=Bu$$

where

(33) $$B=\left[\begin{array}{cccc} cos(\alpha_{1}) & cos(\alpha_{2}) & \cdots & cos(\alpha_{q})\\ sin(\alpha_{1}) & sin(\alpha_{2}) & \cdots & sin(\alpha_{q})\\ L_{1} & L_{2} & \cdots & L_{q} \end{array}\right]$$

In Formula (32), $\tau_{c}\in R^{3}$ is the input control vector, and $u\in R^{q}$ is the vector of the set forces for each actuator.

In control allocation, the optimization of a given quality indicator, such as the minimum energy expenditure for control, is often performed. The problem of optimal control allocation has been considered as a minimization problem with constrained equality [122]

(34) $$\min_{u}u^{T}Wu\;\;in\;relation\;to\;\;Bu=\tau_{c}$$

where $W\in R^{qxq}$ is the weight matrix.

Considering the Lagrangian function, the formulated optimization problem (34) can be written as the unlimited minimization problem in the form

(35) $$L\left(u,\lambda \right)=u^{T}Wu+\lambda^{T}\left(-Bu+\tau_{c}\right)$$

where $\lambda \in R^{3}$ is the vector containing Lagrange multipliers. The Karush–Kuhn–Tucker (KKT) conditions provide the necessary conditions for the optimal solution

(36) $$\frac{\partial L(u,\lambda )}{du}=2Wu-B^{T}\lambda =0\;\;\to \;\;u=\frac{1}{2}W^{-1}B^{T}\lambda$$

(37) $$\frac{\partial L(u,\lambda )}{du}=-Bu+\tau_{c}=0\;\;\to \;\;Bu=\tau_{c}$$

After solving the system of Equations (36) and (37), the sought vector of Lagrange multipliers is obtained as

(38) $$\lambda =2{\left({BW}^{-1}B^{T}\right)}^{-1}\tau_{c}$$

Substituting Equation (38) to (36) gives

(39) $$u=W^{-1}B^{T}{\left({BW}^{-1}B^{T}\right)}^{-1}\tau_{c}$$

When the matrix ${BW}^{-1}B^{T}$ is non-singular, the optimization problem (34) can be solved by finding the solution to the linear equation described by Formula (39).

## 4. Simulations

To demonstrate the effectiveness of the developed control algorithm, selected simulation tests were carried out with the dynamic positioning system.

### 4.1. Ship Model

The mathematical model of *CyberShip I* was chosen as the ship model for determining and testing the control system at low speed. This model was developed in the Department of Engineering Cybernetics, Norwegian University of Science and Technology (NTNU), Trondheim, Norway. The physical model of this ship sails in the Marine Cybernetics Laboratory, NTNU (http://www.ntnu.edu/imt/lab/cybernetics (accessed on 10 September 2021)).

*CyberShip I* is a thruster-controlled model of an offshore supply vessel, made at a scale of 1:70. Its mass is *m* = 17.6 kg with length *L* = 1.19 m. The centre of gravity is located at $x_{G}=-0.04$ m aft of the midship. This point was assumed to be the origin of the body-fixed coordinate system. In its general form, the mathematical model of *CyberShip I* is described by Formula (5). Based on hydrodynamic methods and system identification, model parameters for 3 degrees of *CyberShip I* freedom of motion were found [17,129,130]. The inertia matrix including zero-frequency hydrodynamic added inertia is as follows:

(40) $$M=\left[\begin{array}{ccc} 19.0 & 0 & 0\\ 0 & 35.2 & -0.704\\ 0 & -0.704 & 1.98 \end{array}\right]$$

The matrix of linear interactions related to hydrodynamic damping is defined as

(41) $$D=\left[\begin{array}{ccc} 4 & 0 & 0\\ 0 & 6 & 0\\ 0 & 0 & 1 \end{array}\right]$$

In real-time control systems, the mathematical model of the plant does not fully reflect its dynamics. To bring the simulation tests closer to the conditions in real-time control systems, new parameters of matrices M and D were determined for the model of ship dynamics. In Figure 2, the model of the plant is included in a block labeled “vessel dynamics”. The principle of determining new parameter values was based on the values contained in the model described by matrices (40) and (41). The correction values *Δ* were determined randomly and could at most amount to ±50% of the parameter values included in these matrices. In this way, the following matrix values were determined:

(42) $$M_{vessel}=\left[\begin{array}{ccc} 26.4272 & 0 & 0\\ 0 & 51.3671 & -0.7372\\ 0 & -0.7372 & 1.2645 \end{array}\right]$$

(43) $$D_{vessel}=\left[\begin{array}{ccc} 4.3411 & 0 & 0\\ 0 & 6.2983 & 0\\ 0 & 0 & 1.2577 \end{array}\right]$$

**Figure 3:**
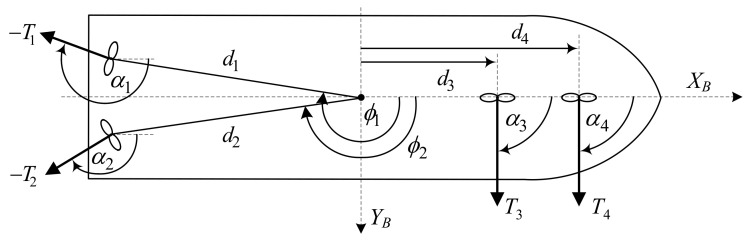
Thruster configuration on *CyberShip I*: *α*_1_ = − 43°, *α*_2_ = − 43°, *α*_3_ = *α*_4_ − 90°, *ϕ*_1_ = 186°, *ϕ*_2_ = 174°, *ϕ*_3_ = *ϕ*_4_, = 0°, *d*_1_ = *d*_2_ = 0.4391 m, *d*_3_ = 0.34 m, *d*_4_ = 0.46 m.

The model of *Cybership I* is equipped with four rpm-controlled thrusters with independently controllable azimuth angles $\alpha_{i}$. The thrusters are controlled by the rotational speed $\omega_{i}$. Figure 3 shows the location of the actuators installed on *CyberShip I*.

The forces and moment generated by the thrusters are given by the formula

(44) $$\tau_{c}=B\left(\alpha \right)T$$

where $B\in R^{3\times 4}$ is the thruster configuration matrix described by Formula (33), in which

(45) $$\begin{array}{c} l_{i,x}=d_{i}cos\left(\phi_{i}\right)\\ l_{i,y}=d_{i}sin\left(\phi_{i}\right) \end{array}\;i\in \left[1,4\right]$$

while the components of the thrust vector of thrusters $T\in R^{4}$ are described by the formula

(46) $$T_{i}=\left\{\begin{array}{cc} k_{iT}\omega_{i}^{2} & \omega_{i}\geq 0\\ k_{iT}\left|\omega_{i}\right|\omega_{i} & \omega_{i}<0 \end{array}\;i\in \left[1,4\right]\right.$$

where $k_{1T}=k_{2T}=k_{4T}=3.125\cdot 10^{-3}$, $k_{3T}=2.5\cdot 10^{-4}$. The thrusts generated by the azimuth thrusters are [131]

(47) $$T_{max}=-T_{min}={\left[0.8\;0.8\;0.1\;0.8\right]}^{T}$$

The inverse characteristics of the thrusters, determined based on Formula (46), have the form

(48) $$\omega_{i}=sgn\left(T_{i}\right)\sqrt{\frac{|T_{i}|}{k_{iT}}}\;i\in \left[1,4\right]$$

The limits imposed on the rotational speeds of thrusters were determined based on Formula (48) and related parameters as

(49) $$\omega_{max}=-\omega_{min}={\left[16\;16\;20\;16\right]}^{T}$$

After taking the related data, the configuration matrix described by Formula (33) takes the form

(50) $$B={\left[\begin{array}{cccc} 0.7314 & 0.7314 & 0 & 0\\ -0.6820 & 0.6820 & 1 & 1\\ 0.3314 & -0.3314 & 0.34 & 0.46 \end{array}\right]}^{T}$$

The limits imposed on the values of forces and moments generated by the thrusters are as follows:

(51) $$\tau_{max}=-\tau_{min}={BT}_{max}={\left[1.17\;0.9\;0.4\right]}^{T}$$

### 4.2. Parameters of Control Systems

The mathematical model of the control system is shown in Figure 2. Here, the vessel model marked as “vessel dynamics” and described by Formula (5) contains the matrix coefficients M and D, given by Formulas (42) and (43), respectively. The coefficients of matrix C are determined from Formula (8). The tested control algorithms described by Formulas (19) and (22) also contain matrices M, C and D. In this case, the values of coefficients described by Formulas (40) and (41) were adopted. Other parameters of the controller determined by the backstepping method (19) are as follows: $K_{0}$ = diag {2, 2, 2}, $K_{1}$ = diag {0.05, 0.05, 0.05}, $K_{2}$ = diag {1, 1, 1}. The parameters of the gain matrices $K_{P}$, $K_{I}$ and $K_{D}$ for the nonlinear PID controller (22) were determined based on the mathematical model of *Cybership I*, given by Formulas (40) and (41), using the Particle Swarm Optimization algorithm and assuming no external disturbances. In this way, the following parameters were obtained for the nonlinear PID controller: $K_{P}$ = diag {1.25, 7.4, 9.95}, $K_{I}$ = diag {0.035, 0.343, 1.0}, $K_{D}$ = diag {3.57, 6.88, 6.06}.

The parameters of the reference model (10) were $\zeta_{i}$ = 1, $\omega_{ni}$ = 0.08, where *i* = 1, 2, 3. The maximum values in Formulas (19) and (22) were imposed as limits on the control signal generated by the DP controllers (51). In the control allocation system, the values of the weight matrix W were W = diag {1, 1, 10, 1}.

The analyzed case concerns dynamic positioning in which the vessel keeps a constant initial position $\eta_{B}$ = [1 m, 1 m, 45°]^T^ for 100 s, then moves to the new position $\eta_{E}$ = [2 m, 1.2 m, °]^T^, to maintain it for the next period of time. The initial conditions of the vessel variable vector were η = [1 m, 1 m, 45°]^T^, ν = [0 m/s, 0 m/s, 0°/*s*]^T^. For the disturbance observer, the initial conditions were $\hat{b}$ = [0 N, 0 N, 0 Nm]^T^.

### 4.3. Case 1—Constant Disturbances

The first analyzed case concerns the situation in which environmental disturbances remained constant in the entire analyzed range of dynamic position control:

(52) $$b\left(t\right)={\left[0.35\;N\;0.35\;N\;0.35Nm\right]}^{T}$$

**Figure 4:**
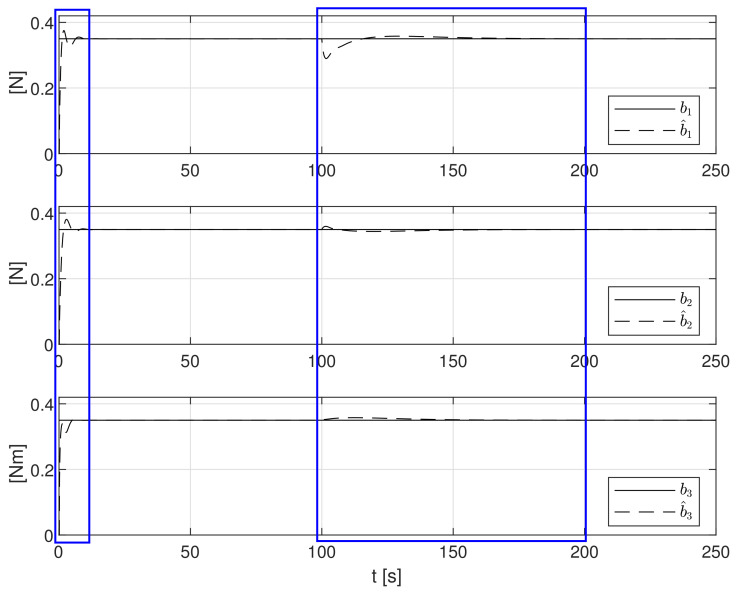
Constant external bias terms *b*_1_, *b*_2_, *b*_3_ and their estimates ${\hat{b}}_{1}$, ${\hat{b}}_{2}$, ${\hat{b}}_{3}$.

**Figure 5:**
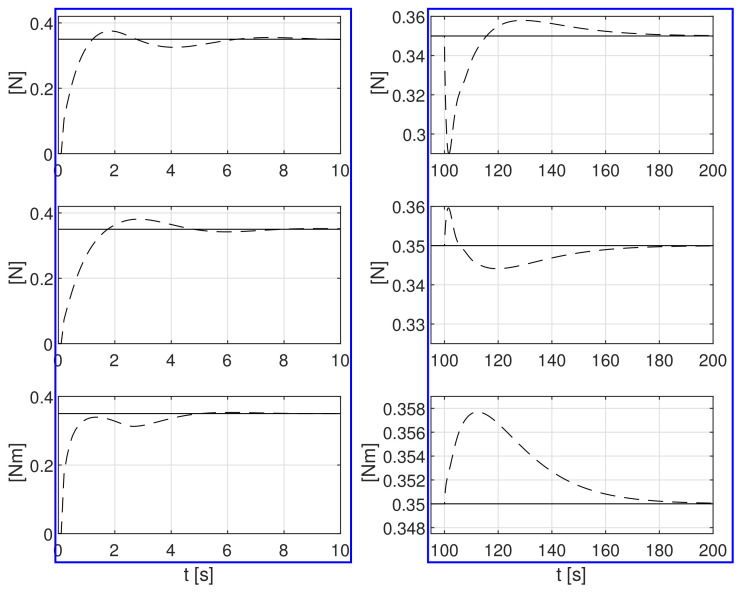
Zoomed partitions of constant external bias terms *b*_1_, *b*_2_, *b*_3_ and their estimates ${\hat{b}}_{1}$, ${\hat{b}}_{2}$, ${\hat{b}}_{3}$ from Figure 4.

**Figure 6:**
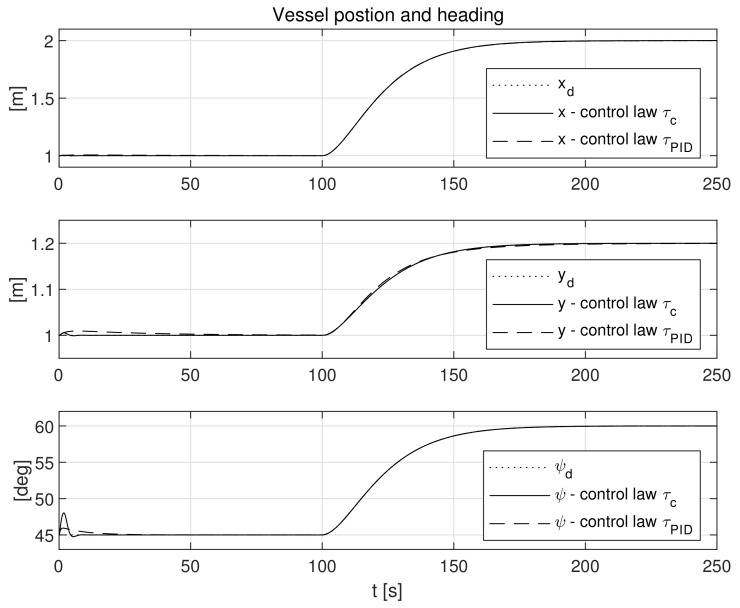
Vessel position (*x*, *y*) and heading (*ψ*) in the presence of constant environmental disturbances.

**Figure 7:**
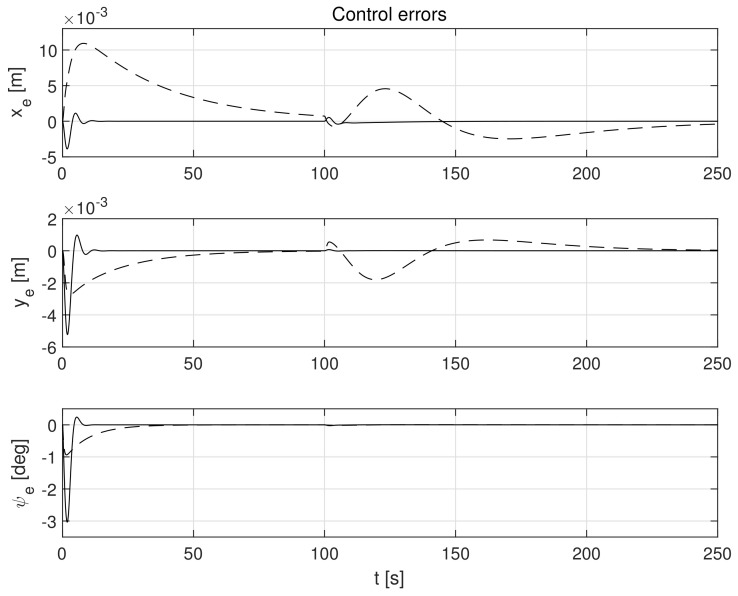
Errors in control systems in the presence of constant environmental disturbances: *x_e_*, *y_e_*-position errors, *ψ_e_*-heading error (solid lines-control law *τ_c_*, dotted lines-control law *τ_PID_*).

**Figure 8:**
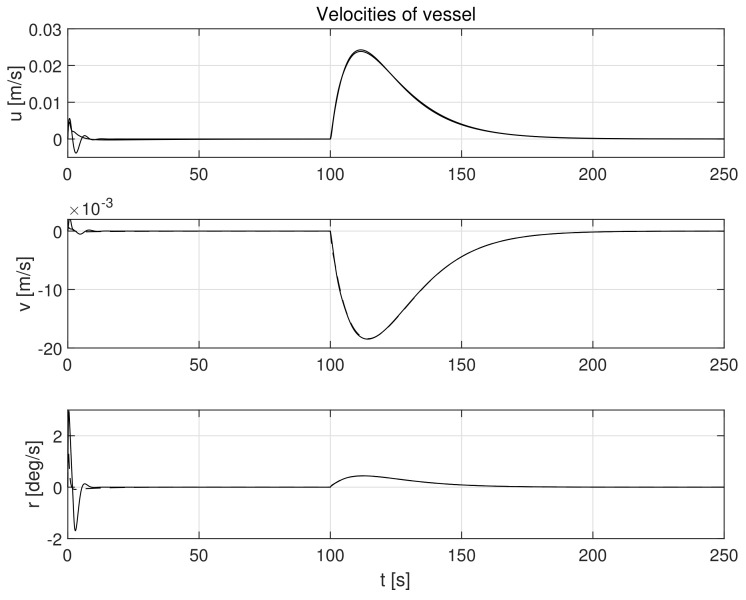
Velocities in control systems in the presence of constant environmental disturbances in surge (*u*), sway (*v*), and yaw (*r*) directions (solid lines-control law *τ_c_*, dotted lines-control law *τ_PID_*).

**Figure 9:**
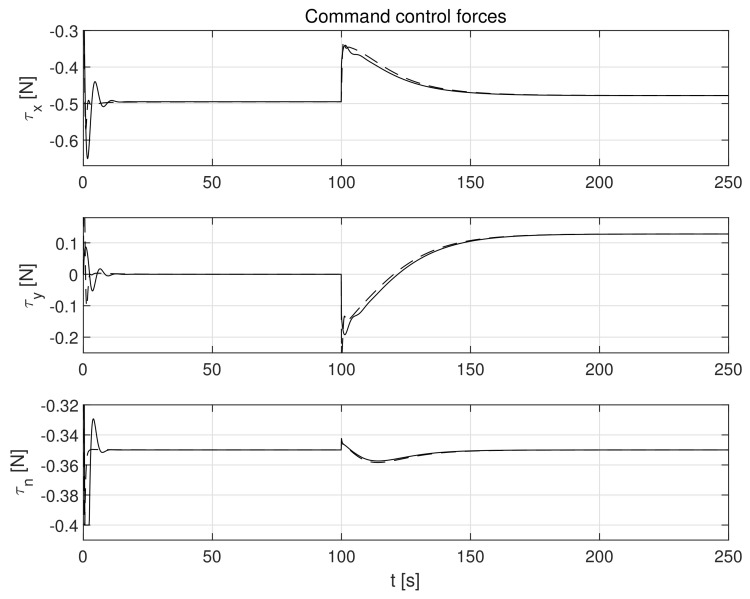
Command forces *τ_x_*, *τ_y_* and moment *τ_n_* at outputs of controllers in the presence of constant environmental disturbances (solid lines-control law *τ_c_*, dotted lines-control law *τ_PID_*).

**Figure 10:**
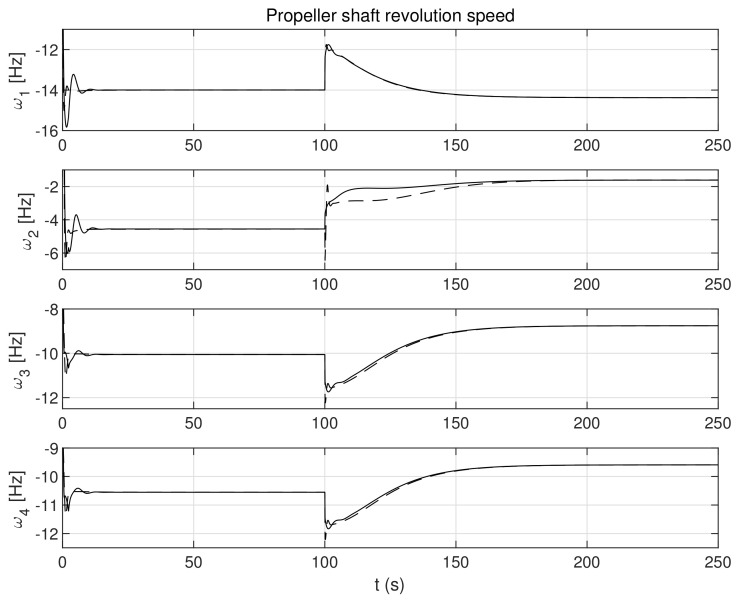
Command propeller shaft revolution speeds in the control systems in the presence of constant environmental disturbances (solid lines-control law *τ_c_*, dotted lines-control law *τ_PID_*).

The simulation test results obtained for this case are shown in Figure 4, Figure 5, Figure 6, Figure 7, Figure 8, Figure 9 and Figure 10. The time histories of environmental disturbances b and their estimates $\hat{b}$ are shown in Figure 4, from which it can be seen that in steady states the disturbance estimates coincide. There are, however, two transients: the first after switching on the control and the second after starting to change the vessel position. The zoomed time segments representing transient states are shown in Figure 5. The first transient lasts about 8 s, while the second is longer and lasts about 80 s. In this latter transient state, the deviations from the real value are very small. Figure 6 shows that the proposed controller is able to follow the desired reference trajectory. The time histories showing the desired and actual ship positions (*x*, *y*) and courses ψ can follow the desired trajectory $\eta_{d}={\left[x_{d},y_{d},\psi_{d}\right]}^{T}$ with good precision. The deviations from the desired values are shown in Figure 7. It is noteworthy that greater values of deviations were recorded in the initial period of time and during the stabilization of the set exchange rate. This is mainly due to the low power of the actuators installed in the *CyberShip I* hull to generate the angular moment $\tau_{n}$. Figure 8 presents the time histories of ship velocity changes in the surge *u*, sway *v* and yaw *r* directions, while the next graphs show the time histories of other quantities occurring in the control system, such as the desired forces $\tau_{x}$, $\tau_{y}$ and moment $\tau_{n}$, which are the output signals from the controllers (Figure 9) and the commanded rotational speeds of the thrusters installed in the vessel’s hull (Figure 10).

### 4.4. Case 2—Stochastic and Time-Varying Disturbances

In the next tested case, the disturbances affecting the ship with stochastic and time-varying forces and moment described by Formula (53) were considered:

(53) $$b\left(t\right)=\left[\begin{array}{c} 0.4+0.05sin(0.035t)+0.1sin(0.025t)+0.1sin(0.045t)\;N\\ 0.3+0.3sin(0.05t)\;N\\ 0.2+0.025sin(0.01t)cos(0.03t)\;Nm \end{array}\right]$$

**Figure 11:**
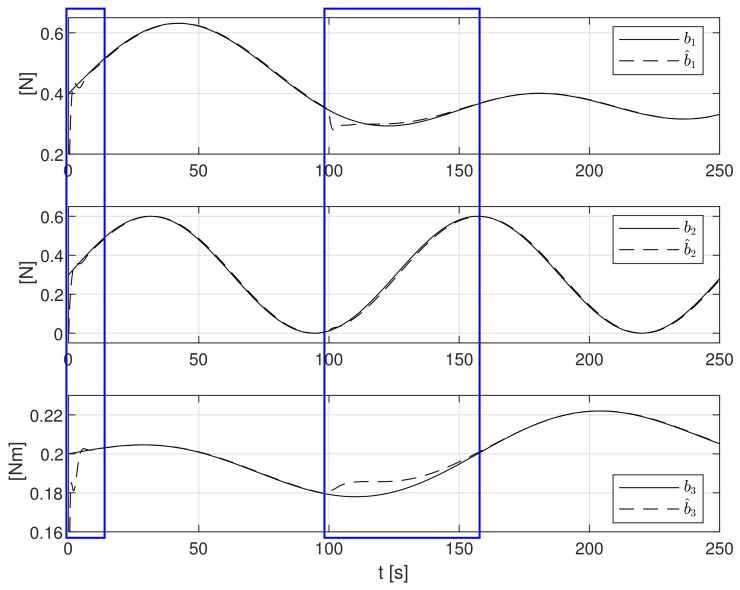
Stochastic and time-varying external bias terms *b*_1_, *b*_2_, *b*_3_ and their estimates ${\hat{b}}_{1}$, ${\hat{b}}_{2}$, ${\hat{b}}_{3}$.

**Figure 12:**
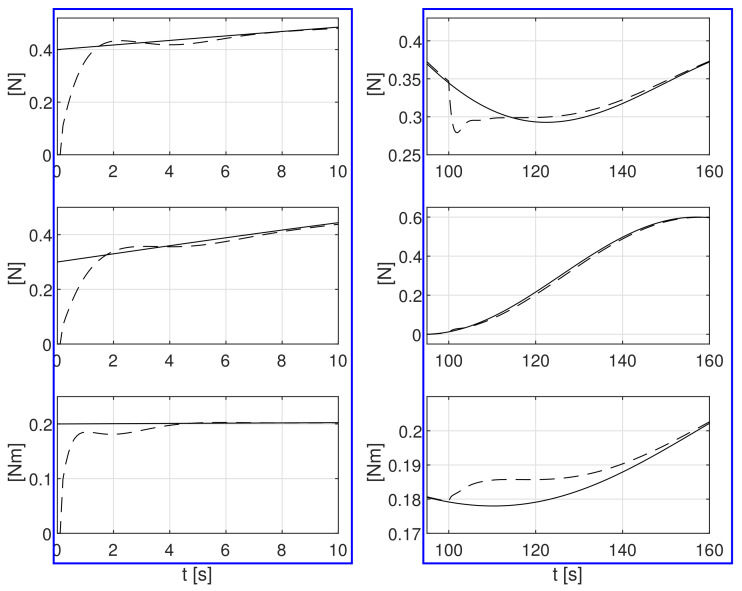
Zoomed partitions of constant external bias terms *b*_1_, *b*_2_, *b*_3_ and their estimates ${\hat{b}}_{1}$, ${\hat{b}}_{2}$, ${\hat{b}}_{3}$ from Figure 11.

**Figure 13:**
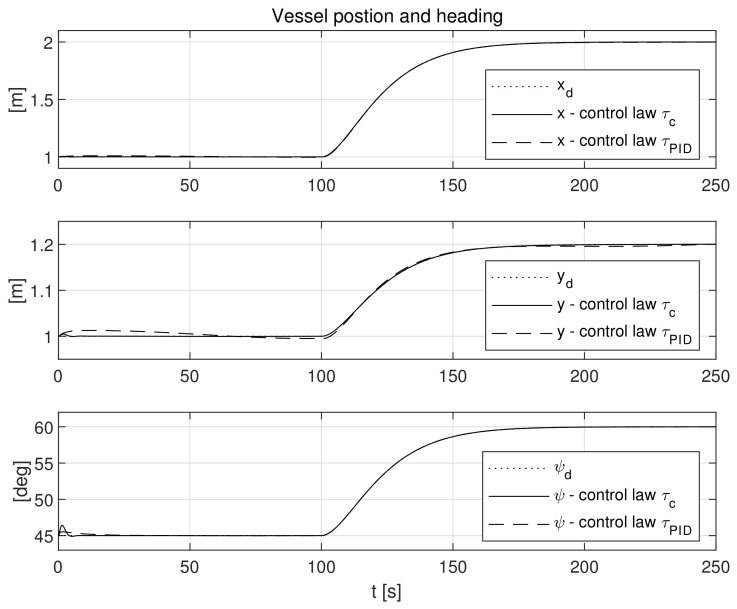
Vessel position (*x*, *y*) and heading (*ψ*) with controllers with stochastic and time-varying disturbances.

**Figure 14:**
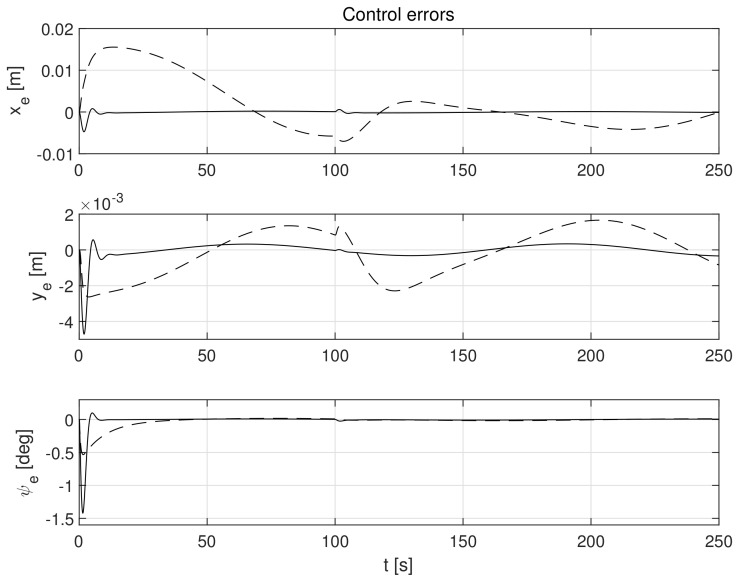
Errors in control systems in the presence of stochastic and time-varying disturbances: *x_e_*, *y_e_*-position errors, *ψ_e_*-heading errors (solid lines-control law *τ_c_*, dotted lines-control law *τ_PID_*).

**Figure 15:**
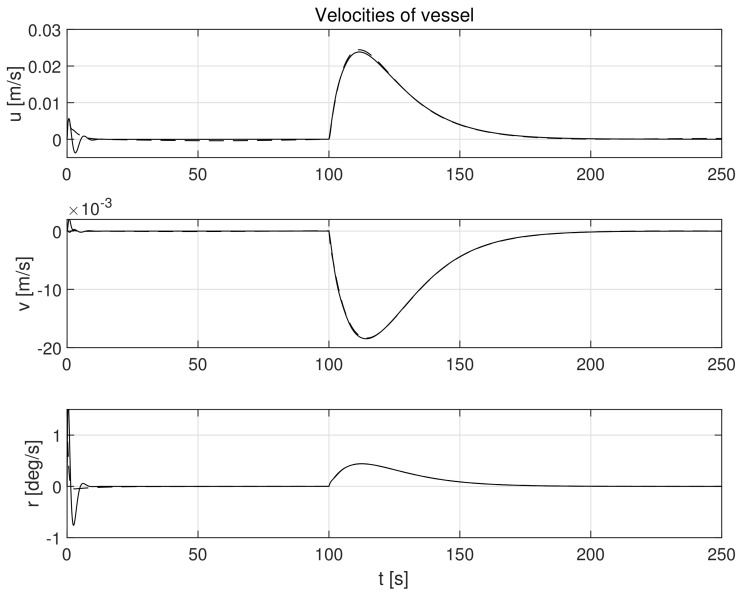
Velocities in control systems in the presence of stochastic and time-varying disturbances: *u*-surge, *v*-sway, *r*-yaw directions (solid lines-control law *τ_c_*, dotted lines-control law *τ_PID_*).

**Figure 16:**
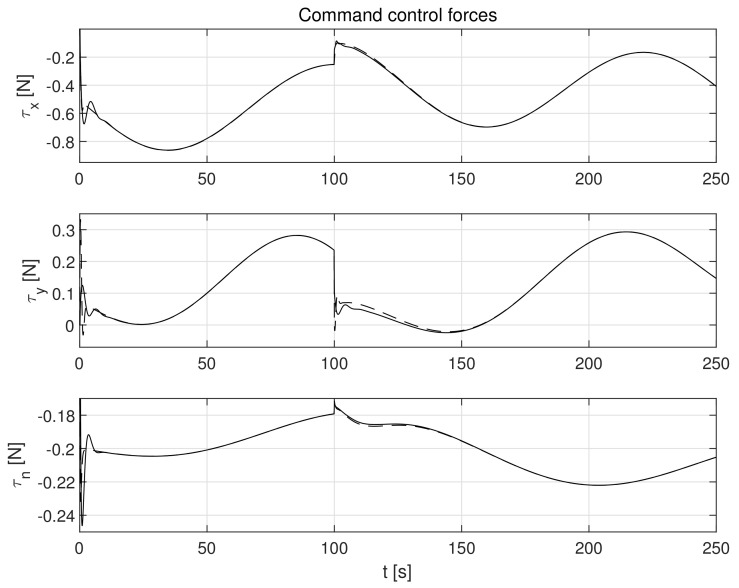
Command forces *τ_x_*, *τ_y_* and moment *τ_n_* in control systems in the presence of of stochastic and time-varying disturbances (solid lines-control law *τ_c_*, dotted lines-control law *τ_PID_*).

**Figure 17:**
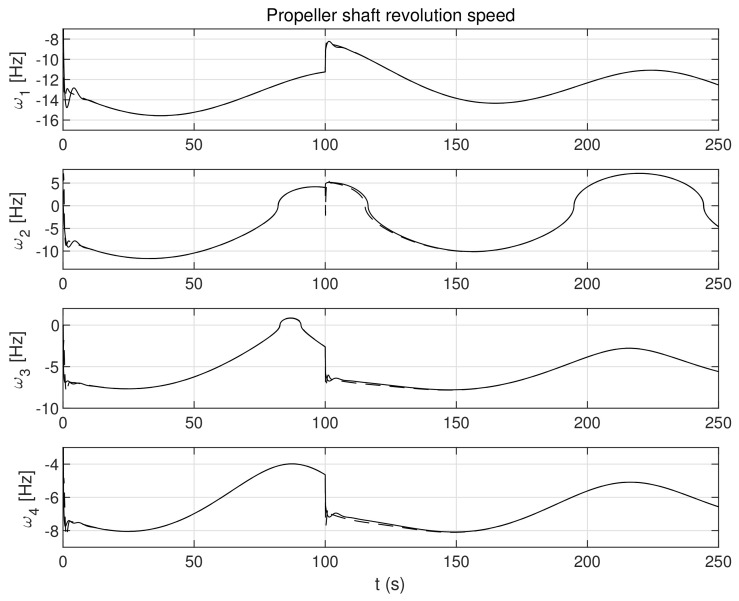
Command propeller shaft revolution speeds in control systems in the presence of stochastic and time-varying disturbances (solid lines-control law *τ_c_*, dotted lines-control law *τ_PID_*).

The simulation test results obtained for this case are shown in Figure 11, Figure 12, Figure 13, Figure 14, Figure 15, Figure 16 and Figure 17. The time histories of environmental disturbances b and their estimates $\hat{b}$ are shown in Figure 11, from which it can be seen that, in the steady states, the disturbance estimates coincide. There are, however, two transients: the first after switching on the control, and the second after starting to change the position of the vessel. The zoomed time segments representing the transient states are shown in Figure 11. The first transient lasts about 10 s, while in the second transient, small deviations are observed in estimates ${\hat{b}}_{1}$ and ${\hat{b}}_{3}$. There are no deviations in the estimate ${\hat{b}}_{2}$. The time histories of the desired and actual ship positions (*x*, *y*) and ship course ψ shown in Figure 13 follow the desired trajectory $\eta_{d}={\left[x_{d},y_{d},\psi_{d}\right]}^{T}$ with good precision. The deviations from the desired values are shown in Figure 14. Figure 15 presents the time histories of ship velocity changes in the surge *u*, sway *v* and yaw *r* directions. The next graphs show the time histories of other quantities occurring in the control system, such as the desired forces $\tau_{x}$, $\tau_{y}$ and moment $\tau_{n}$, which are the output signals from the controllers (Figure 16), and the command rotational speeds of the thrusters installed in the vessel’s hull (Figure 17).

A quantitative comparison of the quality of the performance of the two considered controllers acting in the presence of constant and changing disturbances is given in Table 1, where $x_{e}=x_{d}-x$, $y_{e}=y_{d}-y$ are the errors between the desired and current position, $\psi_{e}=\psi_{d}-\psi$ is the difference between the desired and actual heading of the vessel and $t_{f}$ = 250 s. The results presented in Table 1 clearly show that the controller *τ_c_* (20) has better control quality than the nonlinear *τ_PID_* controller (22).

## 5. Discussion

Numerous institutions and universities are becoming increasingly interested in the development of control algorithms for autonomous marine surface vessels. One of the important tasks is the automation of the process of controlling the surface vessel’s motion for the entire voyage, starting from the departure port and ending at the destination port. In this case, the desired route of the system consists of a number of different-type segments, and thus it may be necessary to use different controllers at different path stages. Ship navigation on the desired route defined in the above way requires the design of a control system that is capable of executing various tasks, such as ship undocking and docking, maneuvering in the port area, movement along the desired route with transit speed and stopping on the route. Such a solution was presented in [132].

The control algorithm presented in this article is planned to be used in a multi-operational system to control the motion of a ship on those segments of the voyage route where the vessel will sail in dynamic positioning mode; i.e., in ports and very narrow navigation canals, on access routes to the port and in the maneuvers performed to reach the docking place.

## 6. Conclusions

The article presents a ship motion control system with a disturbance observer for the dynamic positioning of a fully actuated autonomous marine surface vessel in the presence of uncertain time-variant disturbances due to wind, waves and ocean currents. Both the Coriolis and centripetal matrix and the linear damping matrix are considered in the mathematical model of the vessel. The control strategy is introduced by the backstepping technique with a disturbance observer used to compensate for uncertainties associated with the disturbances. The simulation tests carried out on the model of a sea-going vessel have shown that the designed controller is effective in compensating for external disturbances. The proposed control system is characterized by a good quality of work both during the stabilization of the fixed position of the ship and when moving it to a new position.

The unconstrained control allocation was used to allocate the desired control to individual actuators. The applied allocation method allows for the distribution of the desired forces and moment determined by the DP controller into any number of thrusters installed in the ship’s hull with a fixed thruster, which is a non-rotatable device, and its orientation angle *α* cannot be changed.

## Figures and Tables

**Table 1:** Comparison of quality performance of two control laws *τ_c_* and *τ_PID_* with different disturbances.

Disturbances	Constant		Stochastic and Time-Varying	
Controller	*τ_c_*	*τ_PID_*	*τ_c_*	*τ_PID_*
$\int_{0}^{t_{f}}\left|x_{e}\left(t\right)\right|dt$	0.1948	6.8797	0.4237	11.7068
$\int_{0}^{t_{f}}\left|y_{e}\left(t\right)\right|dt$	0.1564	1.3406	0.6250	2.8474
$\int_{0}^{t_{f}}\left|\psi_{e}\left(t\right)\right|dt$	80.7763	105.9706	45.6141	80.5418

## Data Availability

Data available on request due to restrictions; e.g., privacy or ethical considerations.

## Abbreviations

The following abbreviations are used in this manuscript:

MASS	Maritime Autonomous Surface Ship
DP	Dynamic Positioning
FPSO	Floating Production Storage and Offloading
LQG	Linear Quadratic–Gaussian
NTNU	Norwegian University of Science and Technology
PID	Proportional–Integral–Derivative
